# 9-(4-Chloro­phen­yl)-3,6-diphenyl-1,2,3,4,5,6,7,8-octa­hydro-9*H*-xanthene-1,8-dione

**DOI:** 10.1107/S1600536810015151

**Published:** 2010-04-30

**Authors:** Bin Cui, Yan Jin, Fang-Ming Wang, Li-Zhuang Chen, Guang-Fan Han

**Affiliations:** a School of Material Science and Engineering, Jiangsu University of Science and Technology, Zhenjiang, Jiangsu 212003, People’s Republic of China

## Abstract

In the title compound, C_31_H_25_ClO_3_, the central ring of the xanthene core shows a shallow boat conformation, while the outer six-membered rings display envelope conformations. The dihedral angle between the outer aromatic rings is 88.1 (3)° and the dihedral angles between the chloro­benzene ring and the two phenyl rings are 69.5 (2) and 69.6 (2)°.

## Related literature

For the applications of 3,6,9-tris­ubstituted-1,2,3,4,5,6,7,8-octa­hydroxanthene-1,8(5*H*,9*H*)-dione derivatives, see: Ion *et al.* (1998 ▶); Ahmad *et al.* (2002 ▶); Hunter & Beveridge (2005 ▶); Srihari *et al.* (2008 ▶).
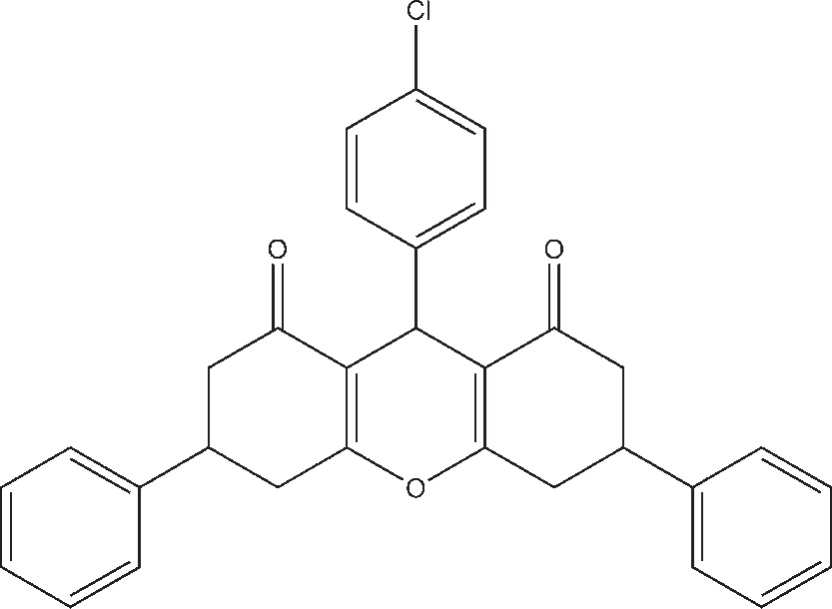


## Experimental

### 

#### Crystal data

C_31_H_25_ClO_3_
*M* *_r_* = 480.96
Orthorhombic, 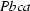

*a* = 9.7591 (14) Å
*b* = 22.133 (3) Å
*c* = 22.290 (3) Å
*V* = 4814.7 (12) Å^3^
*Z* = 8
Mo *K*α radiation
μ = 0.19 mm^−1^
*T* = 291 K
0.25 × 0.20 × 0.10 mm

#### Data collection

Bruker SMART CCD diffractometer
Absorption correction: multi-scan (*SADABS*; Bruker, 2000 ▶) *T*_min_ = 0.955, *T*_max_ = 0.980
24128 measured reflections
4697 independent reflections
2200 reflections with *I* > 2σ(*I*)
*R*_int_ = 0.094

#### Refinement

*R*[*F* ^2^ > 2σ(*F* ^2^)] = 0.088
*wR*(*F* ^2^) = 0.165
*S* = 1.02
4697 reflections
316 parameters
H-atom parameters constrained
Δρ_max_ = 0.22 e Å^−3^
Δρ_min_ = −0.27 e Å^−3^

### 

Data collection: *SMART* (Bruker, 2000 ▶); cell refinement: *SAINT* (Bruker, 2000 ▶); data reduction: *SAINT*; program(s) used to solve structure: *SHELXTL* (Sheldrick, 2008 ▶); program(s) used to refine structure: *SHELXTL*; molecular graphics: *SHELXTL*; software used to prepare material for publication: *SHELXTL*.

## Supplementary Material

Crystal structure: contains datablocks I, global. DOI: 10.1107/S1600536810015151/hb5410sup1.cif

Structure factors: contains datablocks I. DOI: 10.1107/S1600536810015151/hb5410Isup2.hkl

Additional supplementary materials:  crystallographic information; 3D view; checkCIF report

## Figures and Tables

**Fig. 1.**
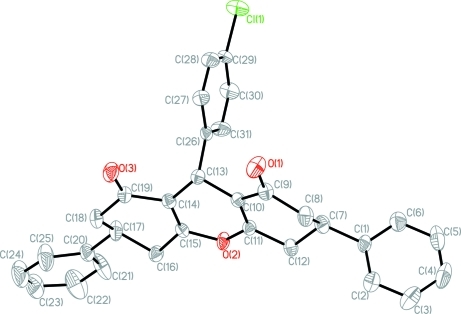
The asymmetric unit of (I) with displacement ellipsoids drawn at the 30% probability level. Hydrogen atoms have been omitted for clarity.

## supplementary crystallographic information

### Comment

3,6,9-trisubstituted-1,2,3,4,5,6,7,8-octahydroxanthene-1,8(5*H*,9*H*)-dione derivatives occupy a prominent position in medicinal chemistry (Ion *et al.*, 1998), and have also been used as laser technology (Ahmad *et al.*, 2002), and as pH-sensitive fluorescent materials (Hunter *et al.*, 2005) and dyes(Srihari *et al.*, 2008). As a contribution in this field, we report herein the crystal structure of the title compound.The title compound (Fig. 1) was synthesized by the condensation reaction of 5-phenyl-1, 3-cyclohexanedione with 4-chlorobenzaldehyde in the presence of dilute H_2_SO_4_ as a catalyst in water. In the xanthene core, the central pyran ring assumes a shallow boat conformation, with atoms C13 and O2 out of the plane through the remaining four atoms [maximum displacement 0.016 (4) Å] by 0.266 (4) and 0.135 (3) Å, respectively. The outer six-membered rings display a half-boat conformation, with atoms C8–C12 and C14–C16/C18/C19 forming a plane [maximum displacement 0.022 (4) and 0.039 (4)] and atoms C7 and C17 displaced by 0.628 (5) and 0.643 (5)Å respectively. The C1–C6, C20–C25 phenyl rings and the C26–C31 benzene ring are tilted with respect to the mean plane through the xanthene core by 79.60 (10)°, 26.34 (13)° and 87.20 (10)° respectively. In the crystal structure, there are weak offset face-to-face π-π stacking interactions with a centroid–centroid distance of 4.1184 (5) Å between the C1–C6 benzene rings and the C26–C31 benzene ring (and the minimum distance among the atoms is C3–C31, that is 3.538 Å)and the dihedral angle between the two benzene rings is 16.418°.

### Experimental

A mixture of a 5-phenyl-1,3-cyclohexanedione (10 mmol, 1.88 g), 4-chloro-benzaldehyde (5 mmol, 0.702 g), and H_2_SO_4_(0.1 ml) in water (40 ml) was stirred at 343-353 K for 2 h. Then the mixture was cooled to room temperature; solid was filtered off and washed with water. The crude products were purified by recrystallization from ethanol (95%). Then the pure products (1 mmol, 0.480 g) were dissolved in the mixtures of 20 ml ethanol and 5 ml *N,N*-dimethylformamide heating to 353 K to form a clear solution and filtering. The filtrate was cooled slowly to room temperature and colourless piece of (I) were formed after 15 days.

### Refinement

All H atoms were placed in calculated positions except H1, H2B, H14A and H14B, with C—H = 0.93-0.98 Å and N—H = 0.86 Å, and refined using a riding model, with U_iso_(H)=1.2U_eq_(C, N, O) or 1.5 U_eq_(C) for methyl H atoms.H1, H2B, H14A and H14B were located in difference fourier maps.

### Crystal data

**Table:**

C_31_H_25_ClO_3_	*F*(000) = 2016
*M**_r_* = 480.96	*D*_x_ = 1.327 Mg m^−^^3^
Orthorhombic, *P**b**c**a*	Mo *K*α radiation, λ = 0.71073 Å
Hall symbol: -P 2ac 2ab	Cell parameters from 1380 reflections
*a* = 9.7591 (14) Å	θ = 2.8–17.6°
*b* = 22.133 (3) Å	µ = 0.19 mm^−^^1^
*c* = 22.290 (3) Å	*T* = 291 K
*V* = 4814.7 (12) Å^3^	Block, colourless
*Z* = 8	0.25 × 0.20 × 0.10 mm

### Data collection

**Table:**

Bruker SMART CCD diffractometer	4697 independent reflections
Radiation source: sealed tube	2200 reflections with *I* > 2σ(*I*)
graphite	*R*_int_ = 0.094
ω scans	θ_max_ = 26.0°, θ_min_ = 1.8°
Absorption correction: multi-scan (*SADABS*; Bruker, 2000)	*h* = −10→12
*T*_min_ = 0.955, *T*_max_ = 0.980	*k* = −27→26
24128 measured reflections	*l* = −26→27

### Refinement

**Table:**

Refinement on *F*^2^	Primary atom site location: structure-invariant direct methods
Least-squares matrix: full	Secondary atom site location: difference Fourier map
*R*[*F*^2^ > 2σ(*F*^2^)] = 0.088	Hydrogen site location: inferred from neighbouring sites
*wR*(*F*^2^) = 0.165	H-atom parameters constrained
*S* = 1.02	*w* = 1/[σ^2^(*F*_o_^2^) + (0.0473*P*)^2^] where *P* = (*F*_o_^2^ + 2*F*_c_^2^)/3
4697 reflections	(Δ/σ)_max_ = 0.008
316 parameters	Δρ_max_ = 0.22 e Å^−^^3^
0 restraints	Δρ_min_ = −0.27 e Å^−^^3^

### Special details

**Table:**

Geometry. All esds (except the esd in the dihedral angle between two l.s. planes) are estimated using the full covariance matrix. The cell esds are taken into account individually in the estimation of esds in distances, angles and torsion angles; correlations between esds in cell parameters are only used when they are defined by crystal symmetry. An approximate (isotropic) treatment of cell esds is used for estimating esds involving l.s. planes.
Refinement. Refinement of *F*^2^ against ALL reflections. The weighted *R*-factor wR and goodness of fit *S* are based on *F*^2^, conventional *R*-factors *R* are based on *F*, with *F* set to zero for negative *F*^2^. The threshold expression of *F*^2^ > σ(*F*^2^) is used only for calculating *R*-factors(gt) etc. and is not relevant to the choice of reflections for refinement. *R*-factors based on *F*^2^ are statistically about twice as large as those based on *F*, and *R*- factors based on ALL data will be even larger.

### Fractional atomic coordinates and isotropic or equivalent isotropic displacement parameters (Å^2^)

**Table:**

	*x*	*y*	*z*	*U*_iso_*/*U*_eq_	
C1	0.5407 (5)	0.39406 (18)	0.3699 (2)	0.0438 (12)	
C2	0.4287 (5)	0.4129 (2)	0.3377 (2)	0.0644 (15)	
H2A	0.3916	0.3870	0.3091	0.077*	
C3	0.3694 (6)	0.4681 (3)	0.3460 (2)	0.0804 (18)	
H3A	0.2936	0.4796	0.3235	0.097*	
C4	0.4240 (7)	0.5065 (2)	0.3883 (3)	0.087 (2)	
H4A	0.3862	0.5446	0.3941	0.104*	
C5	0.5328 (7)	0.4886 (2)	0.4215 (3)	0.0851 (19)	
H5A	0.5676	0.5145	0.4506	0.102*	
C6	0.5935 (5)	0.4326 (2)	0.4132 (2)	0.0625 (14)	
H6A	0.6685	0.4210	0.4362	0.075*	
C7	0.6121 (5)	0.33489 (18)	0.3579 (2)	0.0538 (13)	
H7A	0.6891	0.3330	0.3860	0.065*	
C8	0.6723 (5)	0.33250 (17)	0.29750 (19)	0.0569 (13)	
H8A	0.7278	0.3683	0.2913	0.068*	
H8B	0.5992	0.3329	0.2680	0.068*	
C9	0.7607 (5)	0.27671 (19)	0.28747 (18)	0.0454 (12)	
C10	0.7150 (4)	0.22155 (18)	0.31741 (17)	0.0367 (10)	
C11	0.6030 (4)	0.22346 (18)	0.35110 (17)	0.0376 (11)	
C12	0.5260 (4)	0.27893 (17)	0.36980 (17)	0.0429 (11)	
H12A	0.5041	0.2765	0.4122	0.052*	
H12B	0.4407	0.2815	0.3476	0.052*	
C13	0.7974 (4)	0.16464 (16)	0.31002 (16)	0.0357 (10)	
H13A	0.8289	0.1618	0.2684	0.043*	
C14	0.9226 (4)	0.16727 (16)	0.35157 (17)	0.0342 (10)	
C15	0.9046 (5)	0.16731 (19)	0.41286 (19)	0.0523 (13)	
H15A	0.8165	0.1648	0.4285	0.063*	
C16	1.0139 (5)	0.17096 (19)	0.45124 (19)	0.0548 (13)	
H16A	1.0004	0.1697	0.4925	0.066*	
C17	1.1420 (4)	0.17640 (17)	0.42835 (19)	0.0421 (11)	
C18	1.1638 (4)	0.17601 (18)	0.36775 (19)	0.0499 (12)	
H18A	1.2522	0.1791	0.3525	0.060*	
C19	1.0548 (4)	0.17107 (18)	0.33007 (19)	0.0456 (11)	
H19A	1.0698	0.1702	0.2889	0.055*	
C20	0.7059 (4)	0.11157 (18)	0.32342 (17)	0.0383 (11)	
C21	0.7403 (5)	0.05297 (19)	0.29797 (19)	0.0472 (12)	
C22	0.6491 (5)	0.00033 (18)	0.3145 (2)	0.0583 (13)	
H22A	0.5814	−0.0051	0.2831	0.070*	
H22B	0.7045	−0.0360	0.3160	0.070*	
C23	0.5771 (5)	0.00738 (18)	0.3726 (2)	0.0551 (13)	
H23A	0.6491	0.0112	0.4030	0.066*	
C24	0.4988 (4)	0.06699 (16)	0.37386 (18)	0.0444 (11)	
H24A	0.4206	0.0646	0.3471	0.053*	
H24B	0.4652	0.0746	0.4141	0.053*	
C25	0.5903 (4)	0.11740 (18)	0.35479 (18)	0.0403 (11)	
C26	0.4916 (5)	−0.04687 (19)	0.3914 (2)	0.0517 (13)	
C27	0.4901 (6)	−0.1005 (2)	0.3610 (3)	0.098 (2)	
H27A	0.5363	−0.1036	0.3246	0.117*	
C28	0.4208 (6)	−0.1505 (2)	0.3833 (3)	0.111 (2)	
H28A	0.4232	−0.1866	0.3620	0.133*	
C29	0.3517 (6)	−0.1480 (3)	0.4342 (3)	0.0870 (19)	
H29A	0.3090	−0.1824	0.4493	0.104*	
C30	0.3435 (7)	−0.0948 (3)	0.4644 (2)	0.104 (2)	
H30A	0.2902	−0.0917	0.4989	0.125*	
C31	0.4161 (6)	−0.0448 (2)	0.4431 (2)	0.0828 (19)	
H31A	0.4129	−0.0089	0.4647	0.099*	
Cl1	1.28146 (12)	0.18272 (6)	0.47779 (5)	0.0683 (4)	
O1	0.5432 (3)	0.17227 (11)	0.37532 (11)	0.0434 (8)	
O2	0.8609 (3)	0.27660 (13)	0.25570 (14)	0.0682 (10)	
O3	0.8359 (3)	0.04721 (13)	0.26316 (14)	0.0641 (10)	

### Atomic displacement parameters (Å^2^)

**Table:**

	*U*^11^	*U*^22^	*U*^33^	*U*^12^	*U*^13^	*U*^23^
C1	0.050 (3)	0.028 (3)	0.054 (3)	0.000 (2)	0.008 (3)	0.006 (2)
C2	0.076 (4)	0.052 (3)	0.065 (4)	0.010 (3)	0.004 (3)	−0.005 (3)
C3	0.091 (5)	0.075 (4)	0.075 (4)	0.031 (4)	0.012 (4)	0.015 (3)
C4	0.104 (6)	0.040 (4)	0.117 (6)	0.014 (4)	0.045 (5)	0.002 (4)
C5	0.112 (6)	0.049 (4)	0.094 (5)	−0.015 (4)	0.025 (4)	−0.026 (3)
C6	0.068 (4)	0.052 (3)	0.068 (3)	−0.003 (3)	0.006 (3)	−0.004 (3)
C7	0.059 (3)	0.036 (3)	0.067 (3)	−0.008 (2)	0.004 (3)	0.004 (3)
C8	0.075 (4)	0.035 (3)	0.060 (3)	−0.005 (3)	0.007 (3)	0.013 (2)
C9	0.054 (3)	0.042 (3)	0.041 (3)	−0.005 (3)	0.004 (2)	0.005 (2)
C10	0.040 (3)	0.038 (3)	0.032 (2)	−0.002 (2)	−0.002 (2)	−0.002 (2)
C11	0.042 (3)	0.032 (3)	0.039 (2)	0.000 (2)	0.000 (2)	0.002 (2)
C12	0.054 (3)	0.036 (3)	0.039 (2)	−0.004 (2)	0.004 (2)	0.001 (2)
C13	0.037 (3)	0.039 (3)	0.031 (2)	0.001 (2)	0.006 (2)	−0.001 (2)
C14	0.036 (3)	0.027 (2)	0.040 (2)	−0.004 (2)	0.003 (2)	0.000 (2)
C15	0.036 (3)	0.078 (4)	0.042 (3)	−0.006 (2)	0.008 (2)	−0.001 (3)
C16	0.052 (3)	0.077 (4)	0.036 (3)	−0.007 (3)	0.006 (3)	0.000 (2)
C17	0.034 (3)	0.042 (3)	0.051 (3)	−0.006 (2)	−0.012 (2)	−0.004 (2)
C18	0.039 (3)	0.065 (3)	0.046 (3)	−0.004 (2)	0.010 (2)	−0.002 (3)
C19	0.044 (3)	0.054 (3)	0.039 (2)	−0.001 (2)	0.009 (2)	0.002 (2)
C20	0.042 (3)	0.038 (3)	0.035 (2)	−0.002 (2)	−0.003 (2)	0.001 (2)
C21	0.050 (3)	0.040 (3)	0.052 (3)	0.005 (3)	−0.002 (3)	−0.007 (2)
C22	0.068 (4)	0.032 (3)	0.075 (4)	−0.003 (3)	−0.001 (3)	−0.008 (3)
C23	0.066 (3)	0.034 (3)	0.065 (3)	−0.002 (2)	0.017 (3)	−0.007 (2)
C24	0.045 (3)	0.032 (3)	0.056 (3)	0.001 (2)	0.004 (3)	0.005 (2)
C25	0.042 (3)	0.034 (3)	0.045 (3)	−0.008 (2)	−0.002 (2)	−0.003 (2)
C26	0.052 (3)	0.034 (3)	0.068 (3)	−0.008 (2)	0.005 (3)	0.001 (3)
C27	0.108 (5)	0.055 (4)	0.130 (5)	−0.026 (3)	0.058 (4)	−0.027 (4)
C28	0.108 (6)	0.051 (4)	0.174 (7)	−0.030 (4)	0.046 (5)	−0.021 (4)
C29	0.105 (5)	0.041 (4)	0.115 (5)	−0.022 (3)	0.004 (4)	0.014 (4)
C30	0.160 (7)	0.075 (5)	0.077 (4)	−0.048 (4)	0.013 (4)	0.017 (4)
C31	0.128 (6)	0.056 (4)	0.065 (4)	−0.048 (3)	−0.007 (4)	−0.003 (3)
Cl1	0.0482 (8)	0.0933 (10)	0.0634 (8)	−0.0105 (7)	−0.0103 (7)	0.0026 (7)
O1	0.049 (2)	0.0275 (16)	0.0534 (18)	−0.0016 (14)	0.0189 (15)	0.0001 (14)
O2	0.068 (2)	0.054 (2)	0.083 (2)	0.0053 (18)	0.035 (2)	0.0194 (18)
O3	0.061 (2)	0.052 (2)	0.079 (2)	0.0029 (17)	0.025 (2)	−0.0183 (17)

### Geometric parameters (Å, °)

**Table:**

C1—C2	1.373 (6)	C16—C17	1.355 (5)
C1—C6	1.387 (6)	C16—H16A	0.9300
C1—C7	1.507 (5)	C17—C18	1.367 (5)
C2—C3	1.364 (6)	C17—Cl1	1.757 (4)
C2—H2A	0.9300	C18—C19	1.359 (5)
C3—C4	1.376 (7)	C18—H18A	0.9300
C3—H3A	0.9300	C19—H19A	0.9300
C4—C5	1.353 (7)	C20—C25	1.333 (5)
C4—H4A	0.9300	C20—C21	1.455 (5)
C5—C6	1.387 (6)	C21—O3	1.221 (4)
C5—H5A	0.9300	C21—C22	1.511 (6)
C6—H6A	0.9300	C22—C23	1.484 (5)
C7—C8	1.470 (5)	C22—H22A	0.9700
C7—C12	1.520 (5)	C22—H22B	0.9700
C7—H7A	0.9800	C23—C26	1.520 (5)
C8—C9	1.523 (5)	C23—C24	1.525 (5)
C8—H8A	0.9700	C23—H23A	0.9800
C8—H8B	0.9700	C24—C25	1.491 (5)
C9—O2	1.208 (4)	C24—H24A	0.9700
C9—C10	1.461 (5)	C24—H24B	0.9700
C10—C11	1.327 (5)	C25—O1	1.377 (4)
C10—C13	1.504 (5)	C26—C27	1.367 (6)
C11—O1	1.384 (4)	C26—C31	1.369 (6)
C11—C12	1.499 (5)	C27—C28	1.390 (7)
C12—H12A	0.9700	C27—H27A	0.9300
C12—H12B	0.9700	C28—C29	1.320 (7)
C13—C20	1.506 (5)	C28—H28A	0.9300
C13—C14	1.534 (5)	C29—C30	1.359 (7)
C13—H13A	0.9800	C29—H29A	0.9300
C14—C19	1.379 (5)	C30—C31	1.398 (6)
C14—C15	1.378 (5)	C30—H30A	0.9300
C15—C16	1.370 (5)	C31—H31A	0.9300
C15—H15A	0.9300		
			
C2—C1—C6	118.2 (4)	C17—C16—H16A	120.4
C2—C1—C7	122.6 (4)	C15—C16—H16A	120.4
C6—C1—C7	119.1 (4)	C16—C17—C18	121.0 (4)
C3—C2—C1	122.6 (5)	C16—C17—Cl1	119.0 (3)
C3—C2—H2A	118.7	C18—C17—Cl1	120.0 (4)
C1—C2—H2A	118.7	C19—C18—C17	119.3 (4)
C2—C3—C4	118.8 (6)	C19—C18—H18A	120.4
C2—C3—H3A	120.6	C17—C18—H18A	120.4
C4—C3—H3A	120.6	C18—C19—C14	121.5 (4)
C5—C4—C3	119.9 (6)	C18—C19—H19A	119.3
C5—C4—H4A	120.1	C14—C19—H19A	119.3
C3—C4—H4A	120.1	C25—C20—C21	119.1 (4)
C4—C5—C6	121.6 (6)	C25—C20—C13	122.0 (4)
C4—C5—H5A	119.2	C21—C20—C13	118.7 (4)
C6—C5—H5A	119.2	O3—C21—C20	121.2 (4)
C5—C6—C1	118.9 (5)	O3—C21—C22	121.6 (4)
C5—C6—H6A	120.5	C20—C21—C22	117.2 (4)
C1—C6—H6A	120.5	C23—C22—C21	114.2 (4)
C8—C7—C1	112.2 (4)	C23—C22—H22A	108.7
C8—C7—C12	110.6 (3)	C21—C22—H22A	108.7
C1—C7—C12	115.0 (4)	C23—C22—H22B	108.7
C8—C7—H7A	106.1	C21—C22—H22B	108.7
C1—C7—H7A	106.1	H22A—C22—H22B	107.6
C12—C7—H7A	106.1	C22—C23—C26	114.6 (4)
C7—C8—C9	113.0 (4)	C22—C23—C24	110.1 (4)
C7—C8—H8A	109.0	C26—C23—C24	113.8 (4)
C9—C8—H8A	109.0	C22—C23—H23A	105.8
C7—C8—H8B	109.0	C26—C23—H23A	105.8
C9—C8—H8B	109.0	C24—C23—H23A	105.8
H8A—C8—H8B	107.8	C25—C24—C23	110.0 (4)
O2—C9—C10	120.9 (4)	C25—C24—H24A	109.7
O2—C9—C8	123.1 (4)	C23—C24—H24A	109.7
C10—C9—C8	115.9 (4)	C25—C24—H24B	109.7
C11—C10—C9	118.9 (4)	C23—C24—H24B	109.7
C11—C10—C13	122.0 (4)	H24A—C24—H24B	108.2
C9—C10—C13	119.1 (4)	C20—C25—O1	122.8 (4)
C10—C11—O1	122.8 (4)	C20—C25—C24	125.7 (4)
C10—C11—C12	126.6 (4)	O1—C25—C24	111.4 (4)
O1—C11—C12	110.6 (3)	C27—C26—C31	116.1 (4)
C11—C12—C7	110.0 (4)	C27—C26—C23	123.8 (5)
C11—C12—H12A	109.7	C31—C26—C23	120.0 (4)
C7—C12—H12A	109.7	C26—C27—C28	121.3 (5)
C11—C12—H12B	109.7	C26—C27—H27A	119.4
C7—C12—H12B	109.7	C28—C27—H27A	119.4
H12A—C12—H12B	108.2	C29—C28—C27	121.5 (6)
C10—C13—C20	108.3 (3)	C29—C28—H28A	119.2
C10—C13—C14	109.2 (3)	C27—C28—H28A	119.2
C20—C13—C14	112.5 (3)	C28—C29—C30	119.5 (6)
C10—C13—H13A	108.9	C28—C29—H29A	120.2
C20—C13—H13A	108.9	C30—C29—H29A	120.2
C14—C13—H13A	108.9	C29—C30—C31	119.2 (6)
C19—C14—C15	117.7 (4)	C29—C30—H30A	120.4
C19—C14—C13	122.5 (4)	C31—C30—H30A	120.4
C15—C14—C13	119.8 (4)	C26—C31—C30	122.2 (5)
C16—C15—C14	121.3 (4)	C26—C31—H31A	118.9
C16—C15—H15A	119.3	C30—C31—H31A	118.9
C14—C15—H15A	119.3	C25—O1—C11	116.9 (3)
C17—C16—C15	119.2 (4)		
			
C6—C1—C2—C3	1.1 (7)	C16—C17—C18—C19	1.1 (6)
C7—C1—C2—C3	−175.7 (4)	Cl1—C17—C18—C19	180.0 (3)
C1—C2—C3—C4	0.0 (8)	C17—C18—C19—C14	0.9 (6)
C2—C3—C4—C5	−1.3 (9)	C15—C14—C19—C18	−1.4 (6)
C3—C4—C5—C6	1.4 (9)	C13—C14—C19—C18	177.1 (4)
C4—C5—C6—C1	−0.3 (8)	C10—C13—C20—C25	−19.7 (5)
C2—C1—C6—C5	−1.0 (7)	C14—C13—C20—C25	101.1 (4)
C7—C1—C6—C5	176.0 (4)	C10—C13—C20—C21	156.3 (3)
C2—C1—C7—C8	63.1 (6)	C14—C13—C20—C21	−83.0 (4)
C6—C1—C7—C8	−113.8 (5)	C25—C20—C21—O3	171.2 (4)
C2—C1—C7—C12	−64.5 (6)	C13—C20—C21—O3	−4.9 (6)
C6—C1—C7—C12	118.7 (4)	C25—C20—C21—C22	−6.3 (6)
C1—C7—C8—C9	172.4 (4)	C13—C20—C21—C22	177.6 (3)
C12—C7—C8—C9	−57.8 (5)	O3—C21—C22—C23	156.9 (4)
C7—C8—C9—O2	−147.2 (4)	C20—C21—C22—C23	−25.6 (6)
C7—C8—C9—C10	34.6 (5)	C21—C22—C23—C26	−176.6 (4)
O2—C9—C10—C11	−178.8 (4)	C21—C22—C23—C24	53.5 (5)
C8—C9—C10—C11	−0.6 (6)	C22—C23—C24—C25	−49.5 (5)
O2—C9—C10—C13	2.2 (6)	C26—C23—C24—C25	−179.9 (4)
C8—C9—C10—C13	−179.6 (3)	C21—C20—C25—O1	−173.4 (3)
C9—C10—C11—O1	172.2 (3)	C13—C20—C25—O1	2.5 (6)
C13—C10—C11—O1	−8.9 (6)	C21—C20—C25—C24	8.7 (6)
C9—C10—C11—C12	−9.6 (6)	C13—C20—C25—C24	−175.4 (4)
C13—C10—C11—C12	169.4 (4)	C23—C24—C25—C20	20.0 (6)
C10—C11—C12—C7	−13.8 (6)	C23—C24—C25—O1	−158.1 (3)
O1—C11—C12—C7	164.6 (3)	C22—C23—C26—C27	6.2 (7)
C8—C7—C12—C11	46.5 (5)	C24—C23—C26—C27	134.2 (5)
C1—C7—C12—C11	174.9 (4)	C22—C23—C26—C31	−177.4 (5)
C11—C10—C13—C20	22.8 (5)	C24—C23—C26—C31	−49.4 (6)
C9—C10—C13—C20	−158.3 (3)	C31—C26—C27—C28	−3.0 (9)
C11—C10—C13—C14	−100.0 (4)	C23—C26—C27—C28	173.5 (5)
C9—C10—C13—C14	78.9 (4)	C26—C27—C28—C29	1.3 (11)
C10—C13—C14—C19	−113.0 (4)	C27—C28—C29—C30	2.5 (11)
C20—C13—C14—C19	126.8 (4)	C28—C29—C30—C31	−4.2 (10)
C10—C13—C14—C15	65.4 (5)	C27—C26—C31—C30	1.2 (8)
C20—C13—C14—C15	−54.8 (5)	C23—C26—C31—C30	−175.4 (5)
C19—C14—C15—C16	0.0 (6)	C29—C30—C31—C26	2.4 (9)
C13—C14—C15—C16	−178.5 (4)	C20—C25—O1—C11	14.2 (5)
C14—C15—C16—C17	1.9 (7)	C24—C25—O1—C11	−167.6 (3)
C15—C16—C17—C18	−2.5 (7)	C10—C11—O1—C25	−11.0 (5)
C15—C16—C17—Cl1	178.6 (3)	C12—C11—O1—C25	170.5 (3)
